# Synergy of molecularly mobile polyrotaxane surfaces with endothelial cell co-culture for mesenchymal stem cell mineralization†

**DOI:** 10.1039/d1ra01296g

**Published:** 2021-05-24

**Authors:** Hiroki Masuda, Yoshinori Arisaka, Masahiro Hakariya, Takanori Iwata, Tetsuya Yoda, Nobuhiko Yui

**Affiliations:** Department of Maxillofacial Surgery, Graduate School of Medical and Dental Sciences, Tokyo Medical and Dental University (TMDU) 1-5-45 Yushima Bunkyo Tokyo 113-8549 Japan; Department of Organic Biomaterials, Institute of Biomaterials and Bioengineering, Tokyo Medical and Dental University (TMDU) 2-3-10 Kanda-Surugadai Chiyoda Tokyo 101-0062 Japan yui.org@tmd.ac.jp; Department of Periodontology, Graduate School of Medical and Dental Sciences, Tokyo Medical and Dental University (TMDU) 1-5-45 Yushima Bunkyo Tokyo 113-8549 Japan

## Abstract

Stem cell-based bone tissue engineering is a promising strategy for the treatment of bone defects. Since regeneration of bone tissue takes a long time, promoting osteogenesis of stem cells is desired for earlier recovery from dysfunctions caused by bone defects. Here, we combined endothelial cell co-culture using the molecularly mobile sulfonated polyrotaxane (PRX) surfaces to enhance the mineralization of human bone marrow derived mesenchymal stem cells (HBMSCs). Sulfonated PRXs are composed of sulfopropyl ether-modified α-cyclodextrins (α-CDs) threaded on a polyethylene glycol chain. The molecular mobility of PRX, α-CDs moving along the polymer, can be modulated by the number of α-CDs. When osteoblastic differentiation was induced in HBMSCs and human umbilical vein endothelial cells (HUVECs), co-culture groups on sulfonated PRX surfaces with low molecular mobility showed the highest mineralization, which is about two times as high as co-culture groups on sulfonated PRX surfaces with high molecular mobility. Nuclear accumulation of yes-associated proteins in HBMSCs and cell–cell communication *via* cytokines or cadherin may play an important role in synergistically induced mineralization of HBMSCs.

## Abstract 2

Molecular mobility of polyrotaxane surfaces promoted mineralization in a co-culture system of mesenchymal stem cells and endothelial cells.

## Graphical abstract

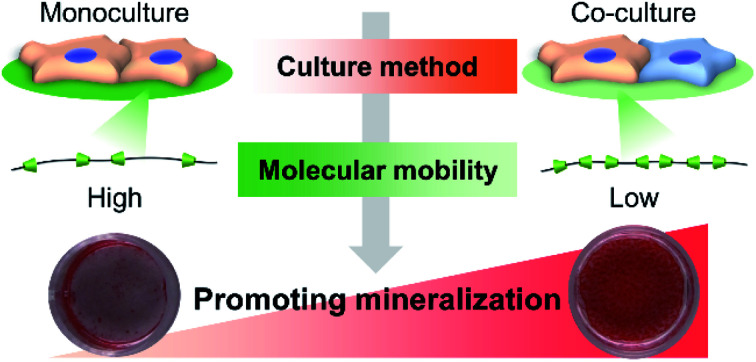

## 1. Introduction

As large-deficiency bone tissues are not spontaneously repaired in the human body, autologous bone,^1^ allogenous bone,^2^ bone prosthetic material,^3^ and growth factors^4^ have been used to reconstruct and regenerate bone tissues. In recent years, mesenchymal stem cells (MSCs), which can differentiate into osteoblasts, have also been applied for regeneration therapy based on bone tissue engineering.^5–7^ Treatment with MSCs has many advantages, such as the spontaneous migration of transplanted MSCs to the injured site,^8^ the suppression of local immune responses,^9^ and the large quantities obtained from the patients themselves.^10^ One of the common challenges in these treatments is to construct an effective scaffold for improving functions of MSCs, particularly osteoblastic differentiation, for bone tissue regeneration.

In the field of biomaterials, the physical characteristics and surface structures of cell adhesive biomaterials that serve as scaffolds are known to play an important role in inducing osteoblastic differentiation and mineralization of MSCs.^11^ For instance, stiff materials promoted the differentiation of MSCs into osteoblasts, compared to soft materials.^12^ As the mechanisms to transmit information of material properties to the cellular nucleus, it has been reported that biological signal pathways related to the Ras homolog gene family, member A (RhoA) and Rho-associated coiled-coil-containing protein kinase (ROCK) activity, yes-associated protein (YAP) activity.^13–16^ The material properties alter the organization of the cytoskeleton through integrins on the cellular membrane.^17^ The organization regulates the activity of RhoA/ROCK and YAP, which are involved in cellular morphology, migration, proliferation, and differentiation.

Previously, we constructed polyrotaxane-based surfaces and succeeded in regulating cellular functions.^18–22^ Polyrotaxane (PRX) is a supermolecule consisting of cyclic molecules, such as α-cyclodextrins (α-CDs), threaded onto an axis polymer, such as poly(ethylene glycol) (PEG).^23^ One of the unique properties of PRX is the molecular mobility, sliding, and rotation of cyclic molecules along the axis polymer. The molecular mobility of PRX can be modulated by changing the number of α-CDs or by changing the functional groups on the α-CDs.^24^ We previously succeeded in regulating the differentiation of MSCs using PRX surfaces with different mobilities.^25^ For instance, the PRX surfaces with low molecular mobility promoted osteoblastic differentiation of MSCs by RhoA activation. Furthermore, the reduced molecular mobility of PRX surfaces was also effective in improving the function of human umbilical vein endothelial cells (HUVECs).^26^ RhoA and YAP activation by molecularly low mobile surfaces contributed proliferation and vascular network formation of HUVECs, which can expect promotion of angiogenesis. Based on these findings, surfaces with low molecular mobility are expected to be suitable for both osteoblastic differentiation of MSCs and the highly angiogenic expression of HUVECs.

In construction of tissue-engineered bone, angiogenesis as well as osteogenesis is essential because inadequate angiogenesis in the implanted bone tissues has a risk of tissue necrosis. For simultaneous facilitation of bone reconstruction and revascularization, it has been reported that co-culture of MSCs with HUVECs is an effective approach for promoting not only osteoblastic differentiation of MSCs but also vascular network formation of HUVECs.^27^ In a co-culture system, osteoblastic differentiation of MSCs was promoted by paracrine effects and direct contact.^28–30^ For instance, morphogenetic protein-2 (BMP-2) secreted from HUVECs promotes mineralization of MSCs.^31^ Simultaneously, vascular endothelial growth factor (VEGF) secreted from MSCs not only enhances proliferation and functions of HUVEC, but also stimulates mineralization of MSCs themselves as autocrine effect for osteogenesis.^32^ In direct contact, expression of neural (N)-cadherin in MSCs was activated by co-culture with HUVECs, resulting in improved cell adhesion and expression of early osteoblastic markers.^28^ Considering these facts, we hypothesize that co-culture of MSCs with HUVECs using PRX surfaces with low mobility may synergistically promote osteoblastic differentiation and mineralization.

In the present study, sulfonated PRX surfaces with different numbers of threaded α-CDs were prepared. To reveal the effect of molecular mobility and co-culture on the osteoblastic differentiation and mineralization of human bone marrow derived MSCs (HBMSCs), alkaline phosphatase (ALP) staining and alizarin red S staining was used. We also examined the gene expression levels of BMP-2, VEGF, neural (N)-cadherin, and type I collagen (COLI) *via* quantitative real-time polymerase chain reaction.

## 2. Experimental

### 2.1 Materials

Sulfonated-PRX triblock copolymers composed of sulfopropyl ether-modified α-CDs threaded onto a PEG chain as a middle PRX segment and poly(benzyl methacrylate) (PBzMA) at both terminals of the PEG as anchoring segments (SPE-PRXs) were prepared as described previously.^33^ SPE-PRXs with different numbers of threading CDs were obtained by altering the PEG/α-CD molar ratios. HBMSCs, an HBMSC Growth Medium BulletKit (HBMSC growth medium), HUVECs, endothelial growth medium-2 (HUVEC growth medium) supplemented with 0.1% VEGF, 0.1% human epidermal growth factor, 0.1% R3-insulin-like growth factor-1, 0.1% ascorbic acid, 0.04% hydrocortisone, 0.4% human fibroblast growth factor-2, 0.1% heparin, 2% fetal bovine serum, and 0.1% gentamicin were purchased from Lonza (Walkersville, MD, USA). Mesenchymal stem cell osteogenic differentiation medium (HBMSC differentiation medium) was purchased from Promo Cell (Heidelberg, Germany). Trypsin/ethylenediaminetetraacetic acid (EDTA) solution, phosphate buffered saline (PBS), 4% paraformaldehyde, alizarin red S, and dimethyl sulfoxide (DMSO) were purchased from FUJIFILM Wako Pure Chemical Corporation (Osaka, Japan). Ammonia solution (28%) was purchased from Kanto Chemical Industry (Tokyo, Japan). A 24-well tissue culture polystyrene (TCPS) plate was purchased from Thermo Fisher Scientific (Waltham, MA, USA).

### 2.2 Fabrication of SPE-PRX surfaces

**Fig. 1:**
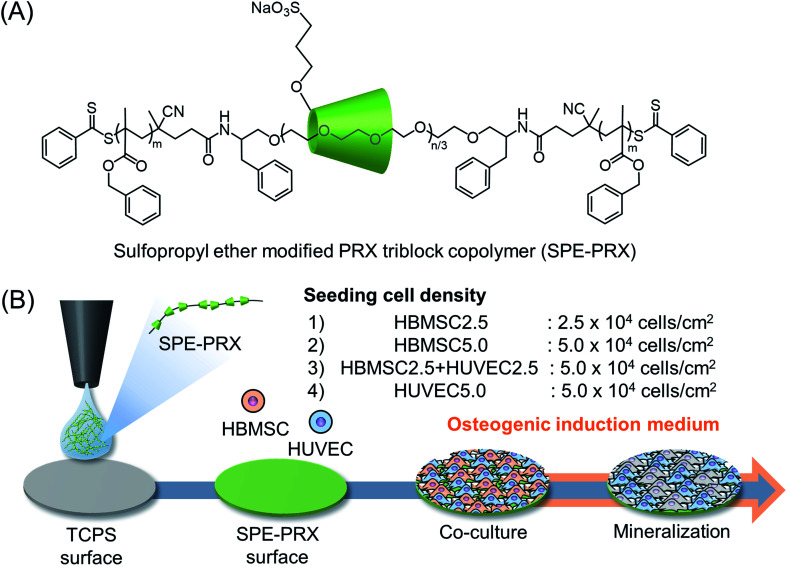
(A) Chemical structure of SPE-PRX. (B) Preparation of SPE-PRX surfaces and mineralization of HBMSCs co-cultured with HUVECs on SPE-PRX surfaces. For induction of osteoblastic differentiation, HBMSCs and HUVECs were seeded on SPE-PRX_5_ or SPE-PRX_86_ surfaces at the following cell densities: HBMSCs at 2.5 × 10^4^ cells per cm^2^ (HBMSC2.5), HBMSCs at 5.0 × 10^4^ cells per cm^2^ (HBMSC5.0), HBMSCs and HUVECs (1 : 1) at total density of 5.0 × 10^4^ cells per cm^2^ (HBMSC2.5 + HUVEC2.5), and HUVECs at 5.0 × 10^4^ cells per cm^2^ (HUVEC5.0).

SPE-PRX copolymers with an α-CD threading number of 5.1 (SPE-PRX_5_) or 86.1 (SPE-PRX_86_) (Fig. 1A) were dissolved in DMSO at a concentration of 0.05 wt%. Next, 30 μL of solution was spread on the 24-well TCPS surfaces and dried at 60 °C for 18 h to obtain SPE-PRX surfaces. All SPE-PRX surfaces were sterilized *via* ultraviolet irradiation for 20 min on a clean bench and washed three times with 500 μL of PBS before the cell experiments.

### 2.3 Cell culture

HBMSCs (Lonza) were cultured in an HBMSC growth medium and used at passage 7. HUVECs (Lonza) were cultured in HUVEC growth medium and used at passage 5–8. For alizarin red S staining and real-time reverse transcription polymerase chain reaction, HBMSCs and HUVECs were seeded on SPE-PRX_5_ or SPE-PRX_86_ surfaces at the following cell densities: HBMSCs at 2.5 × 10^4^ cells per cm^2^ (HBMSC2.5), HBMSCs at 5.0 × 10^4^ cells per cm^2^ (HBMSC5.0), HBMSCs and HUVECs (1 : 1) at a total density of 5.0 × 10^4^ cells per cm^2^ (HBMSC2.5 + HUVEC2.5), and HUVECs at 5.0 × 10^4^ cells per cm^2^ (HUVEC5.0) (Fig. 1B). After 24 h of incubation, the growth medium was replaced with mixed differentiation medium (HBMSC differentiation medium and HUVEC growth medium at a ratio of 1 : 1). The medium was changed every 3 d.

### 2.4 Morphology and proliferation of HBMSCs

HBMSCs were seeded on SPE-PRX_5_ or SPE-PRX_86_ surfaces at a density of 2.0 × 10^3^ cells per cm^2^ and cultured in HBMSC growth medium at 37 °C in a humidified atmosphere with 5% CO_2_ for 6 d. After 24 h of culture, the adhesion area and aspect ratio of cells were analyzed using ImageJ (NIH, Bethesda, MD, USA). The aspect ratio was determined by approximating the cell shape to an ellipse and dividing the long axis by the short axis. At least 29 cells from each surface were analyzed. The cellular density was determined by counting the cells from the captured images at a 1 d interval over 6 d of culture. The adherent cells were observed using a phase contrast microscope (IX71, Olympus) equipped with a dual CCD digital camera (DP80, Olympus). The doubling time of cells on SPE-PRX surfaces was calculated from the change in the number of adherent cells from 72–144 h.

### 2.5 YAP immunostaining of HBMSCs

HBMSCs were seeded on SPE-PRX_5_ or SPE-PRX_86_ surfaces at a density of 2.0 × 10^3^ cells per cm^2^ and cultured in HBMSC growth medium at 37 °C in a humidified atmosphere with 5% CO_2_ for 2 d. Cells were then washed with PBS, fixed in 4% paraformaldehyde at 25 °C for 10 min, and permeabilized with 50 μg mL^−1^ digitonin for 5 min. Cells were washed with PBS and blocked with 3% BSA in PBS for 60 min at 25 °C. Next, the cells were treated with rabbit monoclonal anti-YAP (1 : 1000) primary antibody in 1% BSA in PBS for 18 h at 4 °C. After washing with PBS, cells were treated with Alexa Fluor® 488 goat anti-rabbit IgG H&L (1 : 2000) secondary antibody in 1% BSA in PBS for 60 min at 25 °C. To evaluate subcellular localization, the number of cells representing nuclear and cytoplasmic YAP was counted for more than 140 cells from four samples. Nuclear DNA was stained with Hoechst 33342 (1 : 500). Cells were washed with PBS, and images were acquired using a confocal laser microscope (FV10i, Olympus).

### 2.6 Gene expression analysis

After 7 d of culture, total RNA was isolated using the RNeasy Mini Kit (Qiagen, Valencia, CA, USA) according to the manufacturer's protocol. RNA was suspended in nuclease-free water, and the concentration of RNA was measured using a NanoDrop One/One^c^ spectrophotometer (Thermo Fisher Scientific). Equal quantities of RNA from each sample were reverse-transcribed using a ReverTra Ace qPCR RT master mix (Toyobo, Osaka, Japan) in a T100 thermal cycler (Bio-Rad, Hercules, CA, USA). The reaction conditions were 37 °C for 15 min, 50 °C for 5 min, 98 °C for 5 min, and then, 4 °C for 5 min. Gene expression levels of BMP-2, VEGF, N-cadherin, and COLI relative to the housekeeping gene glyceraldehyde 3-phosphate dehydrogenase (GAPDH) were analyzed using THUNDERBIRD SYBR qPCR mix (Toyobo) on a CFX connect real-time system (Bio-Rad). The primers used for analysis were as follows: BMP-2 (5′-GCCCTTTTCCTCTGGCTGAT-3′ and 5′-TTGACCAACGTCTGAACAATGG-3′), VEGF (5′-AGGAGGAGGGCAGAATCATCA-3′ and 5′-CTCGATTGGATGGCAGTAGCT-3′), N-cadherin (5′-AGTCAACTGCAACCGTGTCT-3′ and 5′-AGCGTTCCTGTTCCACTCAT-3′), COLI (5′-GGAATGAGGAGACTGGCAACC-3′ and 5′-TCAGCACCACCGATGTCCAAA-3′), and GAPDH (5′-CTGACTTCAACAGCGACACC-3′ and 5′-CCCTGTTGCTGTAGCCAAAT-3′) (Life Technologies, Tokyo, Japan). The PCR cycling conditions involved a predenaturation step at 95 °C for 1 min, followed by 40 cycles of 95 °C for 15 s and 60 °C for 1 min as the denaturation step and extension step, respectively. Gene expression levels of BMP-2, VEGF, N-cadherin, and COLI normalized against the housekeeping gene GAPDH were calculated using the 2^−ΔΔ*C*_T_^ method. For levels of gene expression, data are expressed as a fold ratio relative to data acquired for HBMSC2.5 on the SPE-PRX_5_ surface.

### 2.7 Alkaline phosphatase staining

ALP staining was performed after 3, 7, and 14 d of cell culture. Adherent cells were washed with PBS three times and fixed with 4% paraformaldehyde for 20 min at room temperature. The fixed cells were washed twice with Milli-Q water and stained using ALP staining kit (Cosmo Bio, Tokyo, Japan) for 20 min at 37 °C. The stained cells were then washed twice with Milli-Q water.

### 2.8 Alizarin red S staining

Alizarin red S staining was performed after 7, 14, and 21 d of cell culture. First, adherent cells were washed with PBS three times and fixed with 4% paraformaldehyde for 10 min at room temperature. Next, fixed cells were washed twice with Milli-Q water and stained with 500 μL of 1% alizarin red S solution in Milli-Q for 20 min at room temperature. The stained cells were then washed five times with Milli-Q water. Images of cells were acquired using a phase contrast microscope (IX71; Olympus) equipped with a dual CCD digital camera (DP80; Olympus). After imaging, the well plates were allowed to air-dry. After drying, 500 μL of DMSO was added to each well, and the wells were kept under mild shaking for 30 min to completely elute the alizarin red S. Thereafter, 200 μL aliquots of DMSO containing alizarin red S from these wells were obtained, and absorbance was measured at 405 nm using a Varioskan LUX multimode microplate reader (Thermo Fisher Scientific).

### 2.9 Statistical analysis

To assess the significance between data, Student's *t*-test or one-way analysis of variance and *post hoc* analysis using Tukey's range test for multiple comparisons were conducted. All data are expressed as mean ± standard deviation (S.D.).

## 3. Results

### 3.1 Morphology and proliferation of HBMSCs

**Fig. 2:**
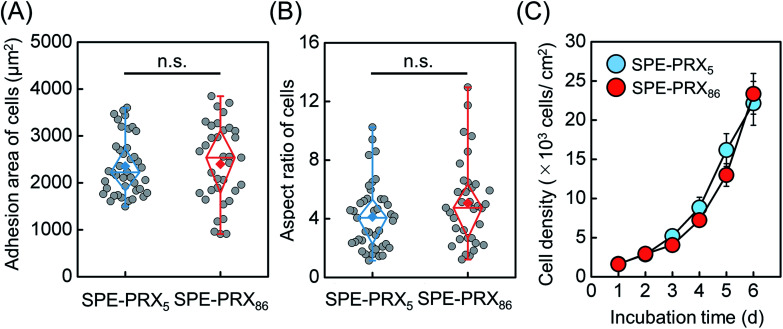
Box plots of the adhesion area (A) and aspect ratio (B) of HBMSCs on SPE-PRX_5_ and SPE-PRX_86_ surfaces. The top and bottom of the boxes correspond to the first and third quartiles. The line in the middle corresponds to the median, the squares represent the mean, and the whiskers represent the maximum and minimum values of data sets. (C) Growth curves of HBMSCs on SPE-PRX_5_ (blue) and SPE-PRX_86_ (orange) surfaces. Data are presented as mean ± S.D., *n* = 4.

When the adhesion area and aspect ratio of HBMSCs on SPE-PRX surfaces were analyzed, the adhesion area of HBMSCs on SPE-PRX_5_ surfaces and SPE-PRX_86_ surfaces were 2950 ± 1160 and 3440 ± 1720 μm^2^, respectively (Fig. 2A and B). The aspect ratio of HBMSCs on SPE-PRX_5_ surfaces and SPE-PRX_86_ surfaces were 4.3 ± 3.2 and 3.8 ± 2.0, respectively. There was no significant difference in the morphology between the SPE-PRX_5_ and SPE-PRX_86_ surfaces. In addition, when HBMSCs were cultured on SPE-PRX_5_ and SPE-PRX_86_ surfaces for 6 d (Fig. 2C), the doubling times of HBMSCs on SPE-PRX_5_ surfaces and SPE-PRX_86_ surfaces were 35.0 ± 4.9 and 28.5 ± 1.1, respectively. The doubling time of cells on SPE-PRX_86_ surfaces tended to be shorter than on SPE-PRX_5_ surfaces.

### 3.2 Subcellular YAP localization of HBMSCs

**Fig. 3:**
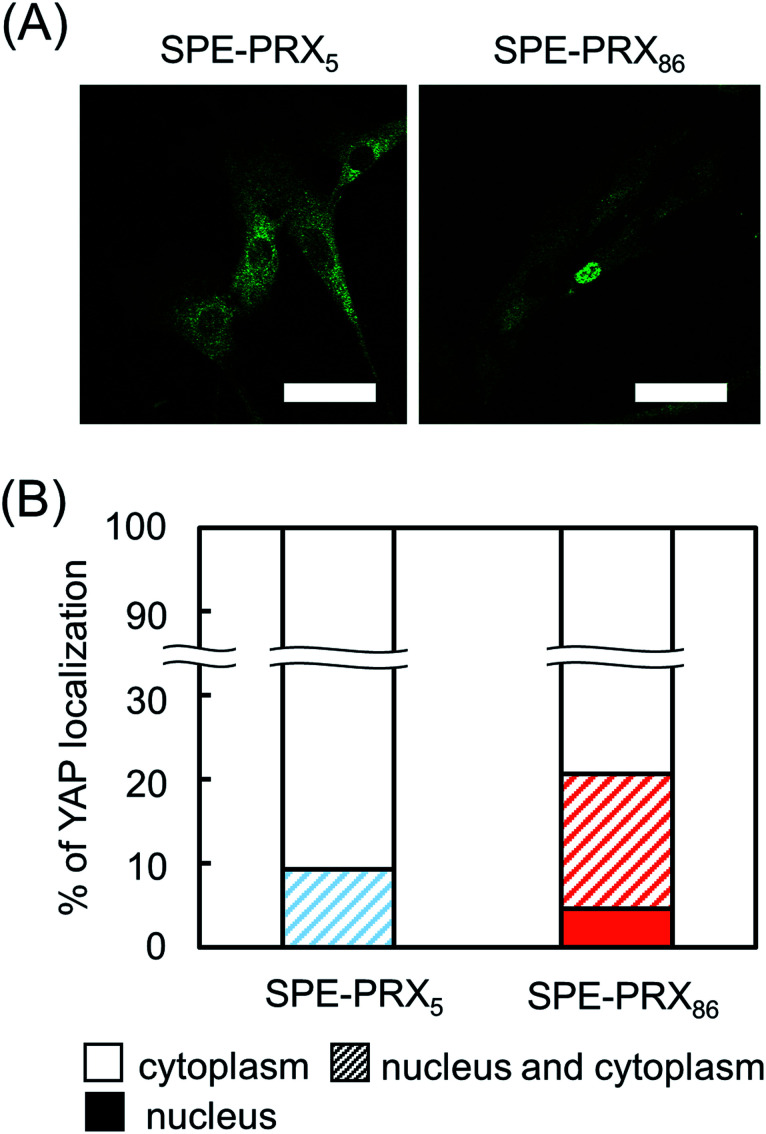
(A) Fluorescent images of YAP localization in HBMSCs on SPE-PRX_5_ and SPE-PRX_86_ surfaces after 2 d of culture. Scale bar: 50 μm. (B) The proportion of YAP localized in nucleus (filled bars), both nucleus and cytoplasm (hatched bars), or cytoplasm (open bars) in HBMSCs.

To analyze the effect of the molecular mobility of SPE-PRXs on nuclear YAP translocation, YAP (green) in adhering HBMSCs on SPE-PRX_5_ and SPE-PRX_86_ surfaces were fluorescently stained (Fig. 3A). On SPE-PRX_86_ surfaces, the proportions of YAP localization in the nucleus only, in both the nucleus and the cytoplasm, and in the cytoplasm only were 4.6%, 16.1%, and 79.3%, respectively (Fig. 3B). In contrast, almost all YAPs in HBMSCs on SPE-PRX_5_ surfaces were localized in both the nucleus and cytoplasm or in the cytoplasm only. The proportions of YAP localization in the nucleus only, in both the nucleus and the cytoplasm and in the cytoplasm only were 0%, 9.3% and 90.7%, respectively (Fig. 3B).

### 3.3 Expression levels of genes related to cell–cell interaction

**Fig. 4:**
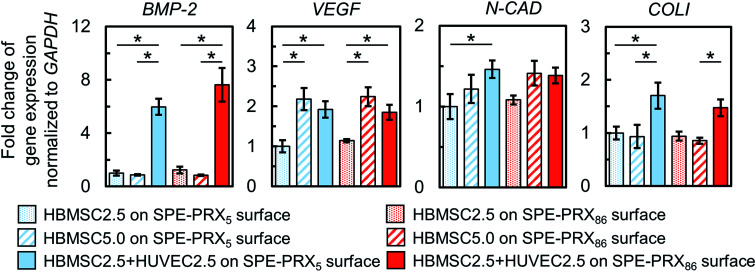
Gene expression levels of BMP-2, VEGF, N-cadherin, and COLI in HBMSCs and HUVECs on SPE-PRX_5_ or SPE-PRX_86_ surfaces after 7 d of culture. Data are presented as mean ± S.D., *n* = 4. Statistical analyses were conducted *via* one-way analysis of variance and *post hoc* analysis using Tukey's range test for multiple comparisons. *p < 0.05 indicates significance.

To investigate the effect of co-culture of HBMSCs and HUVECs on osteoblastic differentiation, gene expression levels of BMP-2, VEGF, N-cadherin, and COLI were quantified on day 7 (Fig. 4). Co-culture with HBMSC2.5 + HUVEC2.5 showed higher expression levels of *BMP-2*, *VEGF*, *N-cadherin*, and *COLI* than monocultures of HBMSC2.5, regardless of the degree of molecular mobility. In particular, HBMSC2.5 + HUVEC2.5 showed higher expression levels of *BMP-2* and *COLI* genes than HBMSC5.0, which has twice the number of MSCs as HBMSC2.5 + HUVEC2.5.

### 3.4 Alkaline phosphatase staining

**Fig. 5:**
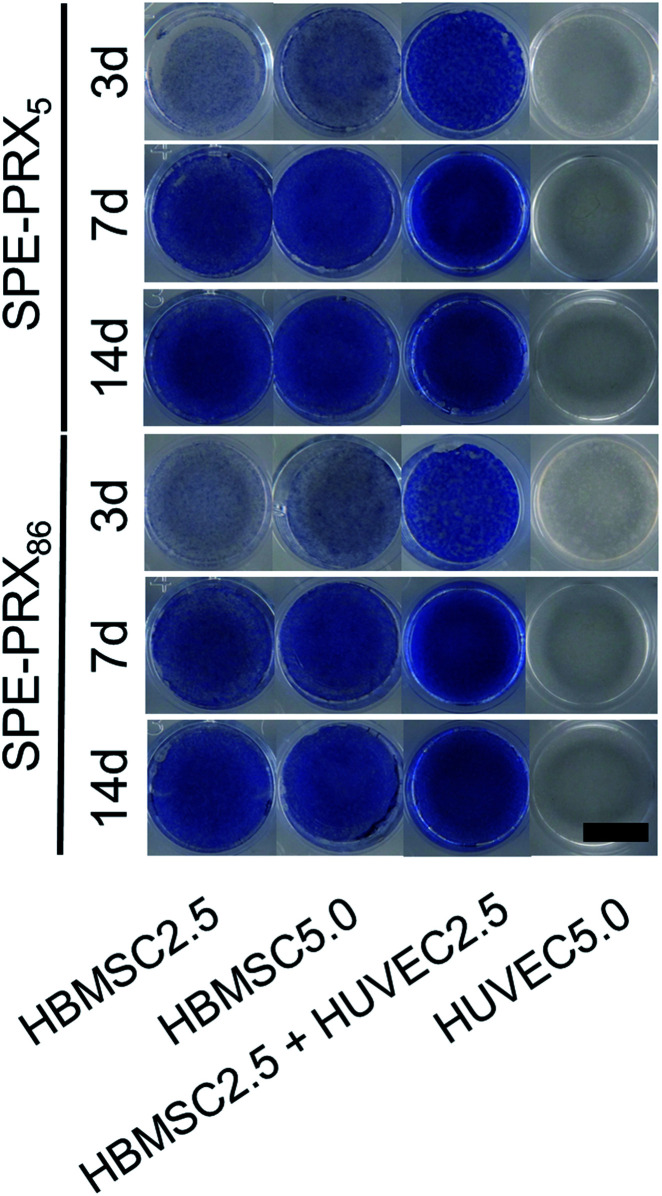
ALP staining images of HBMSCs and HUVECs on SPE-PRX_5_ and SPE-PRX_86_ surfaces after 3, 7, and 14 d of incubation in mixed differentiation medium. Whole well images are shown. Scale bar: 10 mm.

To evaluate the early osteoblastic differentiation of HBMSCs, ALP staining was conducted after 3, 7 and 14 d of cell culture (Fig. 5). Although HUVEC5.0 showed no stainability of ALP, it was observed that HBMSC2.5, HBMMSC5.0 and HBMSC2.5 + HUVEC2.5 showed high stainability of after a 7 d culture. Particularly, HBMSC2.5 + HUVEC2.5 were highly stained within 3 days, suggesting that the co-culture rapidly initiated osteogenic differentiation of HBMSCs, since ALP is an early marker of osteoblastic differentiation.

### 3.5 Quantitative analysis of mineralization by alizarin red S staining

**Fig. 6:**
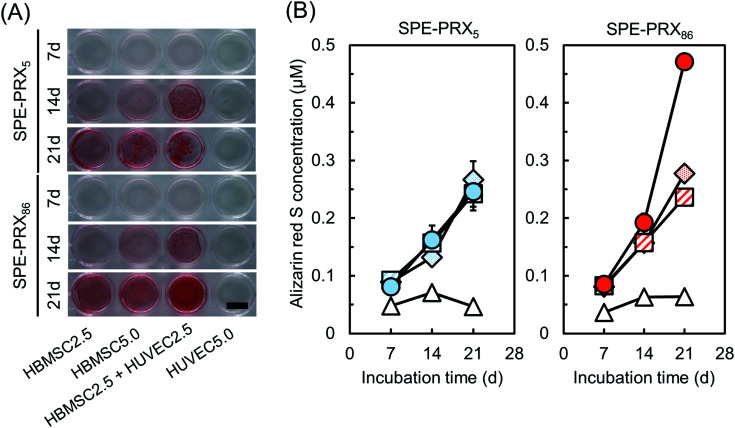
(A) Alizarin red S staining images of HBMSCs and HUVECs on SPE-PRX_5_ and SPE-PRX_86_ surfaces after 7, 14, and 21 d of incubation in mixed differentiation medium. Whole well images are shown. Scale bar: 10 mm. (B) Time courses of alizarin red S concentration of HBMSC2.5 (diamond), HBMSC5.0 (square), HBMSC2.5 + HUVEC2.5 (circle), and HUVEC5.0 (triangle) on SPE-PRX_5_ (blue) surfaces and SPE-PRX_86_ (red) surfaces on day 7, 14, and 21. Data are presented as mean ± S.D., *n* = 4.

Generally, osteoblastic differentiation is demonstrated by mineralization *via* calcium deposition of which degree is measured by alizarin red S staining.^34,35^ To reveal the effect of molecular mobility of SPE-PRX on mineralization, alizarin red S staining was performed after 7, 14, and 21 d of cell culture (Fig. 6 and S1†). Although HUVEC5.0 showed no stainability of alizarin red S, all the groups including HBMSCs showed stainability. HBMSC2.5 + HUVEC2.5 showed higher stainability than HBMSC2.5 on day 21. In addition, HBMSC2.5 + HUVEC2.5 showed higher stainability than HBMSC5.0, even though the number of HBMSCs in HBMSC2.5 + HUVEC2.5 is half as large as HBMSC5.0. Furthermore, HBMSC2.5 + HUVEC2.5 on SPE-PRX_86_ surfaces had higher stainability than on SPE-PRX_5_ surfaces. Although there was no significant difference in alizarin red S concentration between monoculture or co-culture groups on day 7 and 14, HBMSC2.5 + HUVEC2.5 on SPE-PRX_86_ surfaces showed the highest concentration in all groups on day 21. The alizarin red S concentration of HBMSC2.5 + HUVEC2.5 on SPE-PRX_86_ surfaces was approximately two times higher than that of HBMSC2.5 + HUVEC2.5 on SPE-PRX_5_ surfaces on day 21. All these results indicated that co-culture groups using SPE-PRX surfaces with low molecular mobility strongly enhanced mineralization.

## 4. Discussion

**Fig. 7:**
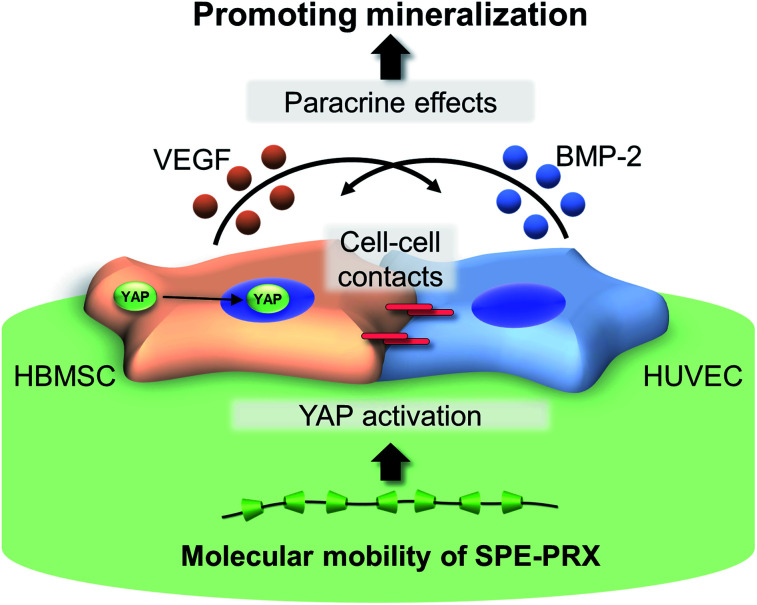
Promoting mineralization of HBMSCs on SPE-PRX surfaces by synergistic effects of translocating YAP to the nucleus by molecular mobility of SPE-PRX and enhanced cell interaction with HUVECs.

In the present study, HBMSC mineralization was significantly enhanced by the synergistic effect of SPE-PRX surfaces with low mobility and co-culture with HUVECs. We considered three major factors involved in enhanced mineralization: (i) subcellular YAP localization in HBMSCs induced by SPE-PRX surfaces with low mobility, (ii) crosstalk with soluble growth factors secreted from HBMSCs and HUVECs, and (iii) cell–cell contacts between HBMSCs and HUVECs. Regarding (i) subcellular YAP localization, the nuclear accumulation of YAP/TAZ promotes cell proliferation and osteoblastic differentiation of MSCs.^36^ Previously, we reported that PRX surfaces with high mobility tend to induce cytoplasmic YAP localization, and PRX surfaces with low mobility tend to induce nuclear YAP localization.^37^ Based on these reports, it is expected that PRX surfaces with low mobility enhance proliferation and osteoblastic differentiation of MSCs more than PRX surfaces with high mobility. In the present study, proliferation and YAP nuclear translocation of HBMSCs on SPE-PRX_86_ surfaces was more facilitated than on SPE-PRX_5_ surfaces, which is consistent with previous reports.^38^ Concerning (ii) soluble growth factors, many studies have reported that co-culture of MSCs and endothelial cells can enhance osteogenic differentiation of MSCs due to the paracrine effects of cytokines (such as BMP-2 and VEGF) produced by each cell.^39–41^ In fact, co-culture of HBMSCs and HUVECs using SPE-PRX surfaces increased the gene expression of BMP-2 and VEGF. The secreted BMP-2 and VEGF was quantitatively confirmed by enzyme-linked immunosorbent assay (Fig. S2†). It has been well known that BMP-2 has an ability to strongly induce osteogenic differentiation *in vitro* and bone regeneration *in vivo*.^29,42^ In the present study, the significant upregulation of BMP-2 gene expression in HBMSC2.5 + HUVEC2.5 on day 7 may have promoted higher mineralization of HBMSC2.5 + HUVEC2.5 on SPE-PRX_86_ surfaces on day 21 than HBMSC2.5 and HBMSC5.0. As for (iii) cell–cell contacts, previous studies reported that N-cadherin-mediated cell–cell contacts may induce intracellular signaling events, leading to osteoblastic gene expression such as COLI.^28,43^ The expression of N-cadherin and COLI in HBMSC2.5 + HUVEC2.5 cells was significantly higher than that in HBMSC2.5. The following three factors (i) translocating YAP to the nucleus by low molecular mobility, (ii) secreted BMP-2 and VEGF, and (iii) enhanced N-cadherin expression may have synergistically enhanced osteoblastic differentiation and mineralization of HBMSCs (Fig. 7).

In the present study, HBMSC2.5 + HUVEC2.5 on SPE-PRX_86_ surfaces on day 21 showed the highest stainability of alizarin red S, an indicator of mineralization, compared to monoculture or co-culture using SPE-PRX_5_ surfaces. It is obvious that synergy of low molecular mobility of SPE-PRX surfaces and co-culture with HUVECs could strongly facilitate the mineralization.

## 5. Conclusions

In the present study, we investigated the effect of the molecular mobility of SPE-PRX surfaces on osteoblastic differentiation of HBMSCs co-cultured with HUVECs. On SPE-PRX surfaces with low mobility, YAP nuclear translocation was promoted in HBMSCs. In addition, co-culture of HBMSCs with HUVECs enhanced osteogenesis and angiogenesis-related gene expression more than monoculture of HBMSCs. Mineralization was strongly induced by the synergistic effects of low molecular mobility of SPE-PRX and co-culture of HBMSCs with HUVECs. Promotion of osteoblastic differentiation and mineralization using PRX-based surfaces would be a powerful tool for facilitating bone regeneration in elderly people or patients with osteoporosis.

## Conflicts of interest

There are no conflicts to declare.

## Supplementary Material

RA-011-D1RA01296G-s001
