# Supplementary material for: Synergy of molecularly mobile polyrotaxane surfaces with endothelial cell co-culture for mesenchymal stem cell mineralization
Source: RSC Adv. 2021 May 24;11(30):18685–92. doi: 10.1039/d1ra01296g (PMC9033494; doi:10.1039/d1ra01296g)
Supplement: RA-011-D1RA01296G-s001 [file RA-011-D1RA01296G-s001.pdf]

Electronic Supplementary Material (ESI) for RSC Advances.
This journal is © The Royal Society of Chemistry 2021

Supporting Information

Synergy of molecularly mobile polyrotaxane surfaces with endothelial cell co-culture for mesenchymal stem cell mineralization

Hiroki Masuda,[†a] Yoshinori Arisaka,[†b] Masahiro Hakariya,[c] Takanori Iwata,[c] Tetsuya Yoda,[a] and Nobuhiko Yui[b]

[a.] Department of Maxillofacial Surgery, Graduate School of Medical and Dental Sciences, Tokyo Medical and Dental University (TMDU), 1-5-45 Yushima, Bunkyo, Tokyo 113-8549, Japan

[b.] Department of Organic Biomaterials, Institute of Biomaterials and Bioengineering, Tokyo Medical and Dental University (TMDU), 2-3-10 Kanda-Surugadai, Chiyoda, Tokyo 101-0062, Japan

[c.] Department of Periodontology, Graduate School of Medical and Dental Sciences, Tokyo Medical and Dental University (TMDU), 1-5-45 Yushima, Bunkyo, Tokyo 113-8549, Japan

† These authors contributed equally to this study.

Alizarin red S staining

To evaluate mineralization, alizarin red S staining was performed on 7, 14 and 21 d of cell culture. Images of cells were acquired using a phase-contrast microscope (IX71; Olympus) equipped with a dual CCD digital camera (DP80; Olympus).

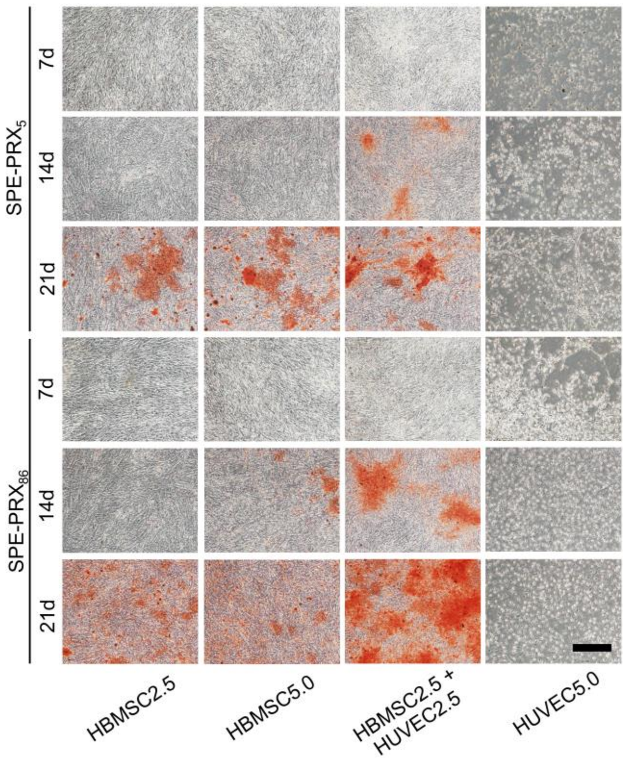

Fig. S1 Phase-contrast microscopic images of alizarin red S staining cells cultured on SPE-PRX$_5$ or SPE-PRX$_{86}$ surfaces after 7, 14 and 21 d of culture. Scale bar: 500 µm.

Quantification of BMP-2 and VEGF secretion

The concentration of BMP-2 and VEGF secreted by cells in the supernatant of HBMSC2.5+HUVEC2.5 were determined by enzyme-linked immunosorbent assay (ELISA). Supernatant was collected after a 7-d culture and was analyzed by a Human BMP-2 ELISA Kit (Thermo Fisher Scientific) and Human VEGF ELISA Kit (Proteintech) according to the protocol provided by the manufacturer. The absorbance was measured at 450 nm using a Varioskan LUX multimode microplate reader (Thermo Fisher Scientific).

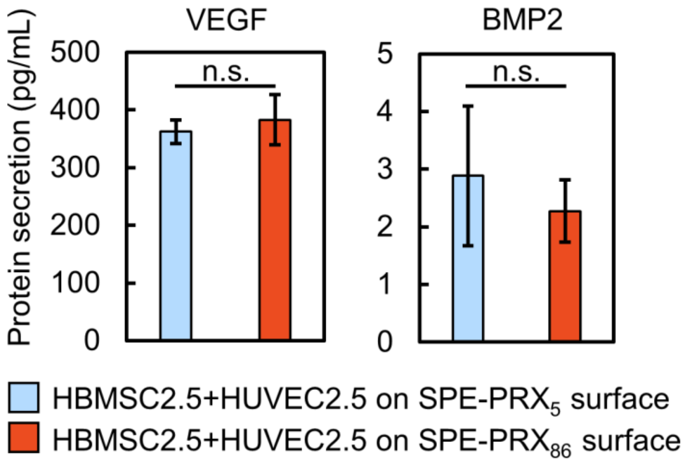

Fig. S2 Quantification of BMP-2 and VEGF secretion on SPE-PRX$_5$ or SPE-PRX$_{86}$ surfaces after 7 d of culture. Data are presented as mean ± S.D., n = 4. Statistical analyses were conducted via one-way analysis of variance and post hoc analysis using Tukey's range test for multiple comparisons. *$p < 0.05$ indicates significance.
